# MSR405: Inhibiting Neuroinflammation after Spinal Cord Injury in Rats

**DOI:** 10.3390/biomedicines12030614

**Published:** 2024-03-08

**Authors:** Yu Liu, Yu Xiao, Jimeng Gao, Jiaxin Gao, Ruicheng Li, Zhongquan Qi, Xiaocun Liu

**Affiliations:** 1 Medical College, Guangxi University, Nanning 530004, China; liuyu_2021_v@126.com (Y.L.); xyrain611@163.com (Y.X.); gjm221106@163.com (J.G.); 2028391005@st.gxu.edu.cn (J.G.); peterli0021@126.com (R.L.); 2 Xiang’an Hospital of Xiamen University, Xiamen 361005, China

**Keywords:** *Radix Isatidis*, MSR405, SCI, anti-inflammation, NF-κB

## Abstract

The treatment of spinal cord injury (SCI) is often ineffective. Additionally, SCI-induced inflammation leads to secondary injury. Current anti-inflammatory hydrophilic drugs fail to reach the nerve injury site due to the blood–brain barrier. Here, we synthesized MSR405, a new lipophilic unsaturated fatty acid derivative of *Radix Isatidis* and investigated its therapeutic effect in SCI model rats. Furthermore, we systematically investigated its structure, toxicity, anti-inflammatory effect, and the underlying mechanism. MSR405 was injected into the abdominal cavity of the Sprague Dawley SCI model rats, and the effect on their behavioral scores and pathology was estimated to assess the status of neurological inflammation. Our data show that MSR405 treatment significantly improved the motor function of SCI rats, and markedly suppressed the associated neuroinflammation. Moreover, MSR405 could attenuate LPS-induced inflammatory response in BV2 cells (Mouse microglia cells) in vitro. Mechanistically, MSR405 inhibits proinflammatory cytokines, supporting the anti-inflammatory response. Additionally, MSR405 can significantly block the TLR4/NF-κB signaling pathway and nitric oxide production. In summary, MSR405 reduces inflammation in SCI rats through the TLR4/NF-κB signal cascade and can inhibit neuroinflammation after spinal cord injury.

## 1. Introduction

Spinal cord injury (SCI) is a severe neurological disorder that often results in enduring impairments of motor, sensory, and autonomic functions [1], involving neuronal damage and inflammation [2]. During the last 30 years, its global prevalence has increased from 236 to 1298 cases per million people [3]. In China, the mean age of SCI cases at the time of injury was between 31.5 and 50.1 years, with a pooled mean age of 45.4 years and the proportion of male patients was higher [4]. The death of neuronal cells and inflammation following SCI leads to the degeneration and loss of neurological functionality [5]. About 3–7 days after SCI, infiltrating microglia reach their peak levels, releasing additional pro-inflammatory cytokines [6], thereby causing secondary damage to the nerve injury site. Therefore, reducing proinflammatory cytokines and increasing the activities of anti-inflammatory cytokine could be a potential therapeutic strategy for SCI [7,8]. Unfortunately, SCI is currently incurable. Patients with SCI require a prolonged and high level of specialized nursing. Treatments only maximize residual function by rehabilitating and minimizing secondary complications [9]. To date, methylprednisolone is the only pharmacological agent that has received clinical approval for the treatment of SCI, but the risks associated with corticosteroid treatment (e.g., gastrointestinal bleeding and wound infection) and limitations in functional recovery restrict its use [10].

After SCI, the proinflammatory cytokines are released, such as tumor necrosis factor-alpha (TNF-α), interleukin-1-Beta (IL-1β), and interleukin-6 (IL-6) [11]. Toll-like receptors (TLRs) can initiate innate immune defense. Among them, Toll-like receptor 4 (TLR4) is one of the most important members of TLR. More and more evidence shows that TLR4 plays a critical role in triggering the inflammation of spinal cord [12]. The activation of TLR4 leads to the release of nuclear factor Kappa B (NF-κB), which in turn provokes many pro-inflammatory mediators including TNF-α, IL-1β, and IL-6 [13].

Small-molecule compounds have the unique advantages of high cell permeability, reversibility, and ease of manipulating cell fate regulation; thus, this approach is a promising new strategy for regulating cell fate [14]. Traditional Chinese medicines, such as ginsenosides, genistein, tanshinone, and *Radix Isatidis*, also mediate neuroprotection and promote neural the function recovery of SCI [14].

*Radix Isatidis*, a prominent traditional Chinese medicinal herb that belongs to the family Brassicaceae, is widely used in China [15]. Numerous studies have reported its remarkable anti-inflammatory properties [16]. However, the potential mechanisms which are responsible for *Radix Isatidis* were not completely indicated. Accordingly, we extracted *Radix Isatidis* unsaturated fatty acids as lead compounds and optimized their structure to develop an acyl urea compound known as N,N′-dicyclohexyl-N-linolenic acid ureide (MSR405). MSR405 has lipophilic properties (long-chain fatty acids), facilitating its passage across the blood–brain barrier, and is recognized for its low toxicity and minimal side effects [17,18,19]. Subsequently, we examined the therapeutic impact of MSR405 on SCI and associated inflammation via TLR4/NF-κB signaling.

In this study, we improved the synthesis method of MSR405 to achieve higher yield and purity. We then investigated the possible effect of MSR405 on the inflammatory response in an SCI rat model and explored the underlying mechanism in the lipopolysaccharide (LPS)-stimulated BV2 cell model. However, further exploration is necessary to fully understand the impact of MSR405 on microglia and its implications for nerve repair following SCI.

## 2. Materials and Methods

### 2.1. MSR405 Synthesis

**Figure 1:**
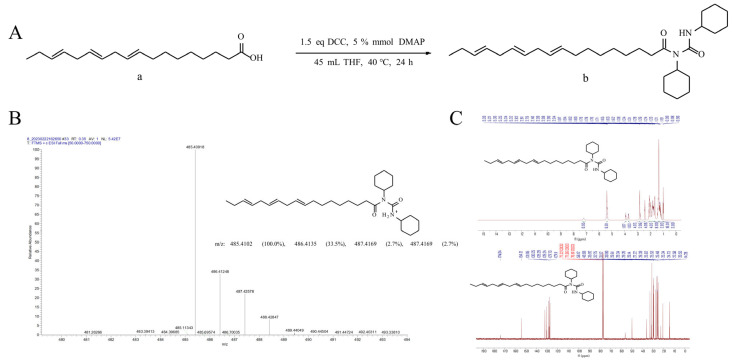
Synthesis and identification of MSR405. (**A**) Synthesis of compounds (**b**). Reagents and conditions: 30 mmol α-linolenic acid (**a**), 45 mmol DCC, 5% mmol DMAP, 45 mL THF, rt, 24 h. (**B**,**C**) (9E,12E,15E) -N-cyclohexyl-N- (cyclohexyl carbamoyl) octadeca-9,12,15-trienamide (**b**). yellow oil, yield 60%. 1 H NMR (500 MHz, CDCl3) δ 7.15 (s, 1H, N-H), 5.36 (ddd, J = 18.4, 11.7, 5.7 Hz, 6H, H-C=C-H), 3.89 (t, J = 12.1 Hz, 1H, -N-CH-), 3.68 (dt, J = 10.4, 3.5 Hz, 1H, -NH-CH-), 2.82–2.76 (m, 4H, -C=C-CH_2_-C=C-), 2.40 (t, J = 7.3 Hz, 2H, -CH_2_CON-), 2.42–2.39 (m, 4H, CH_3_-CH_2_-, -C=C-CH_2_CH_2_-), 2.11–2.03 (m, 4H, -(CH_2_)_5_-, cyclohexyl), 1.83–1.69 (m, 8H, -(CH_2_)_5_-, cyclohexyl), 1.65–1.60 (m, 2H, -(CH_2_)_5_-, cyclohexyl), 1.36–1.21 (m 16H, -(CH_2_)_5_-), cyclohexyl, 0.98 (t, J = 7.6 Hz, 3H, -CH_3_).; 13 C NMR (126 MHz, CDCl_3_) δ 174.04 (N-CO-), 154.12 (N-CO-N), 131.95 (-C=C-), 130.25 (-C=C-), 128.29 (-C=C-), 128.24 (-C=C-), 127.73 (-C=C-), 127.11 (-C=C-), 56.12 (-N-CH-), 49.69 (-NH-CH-), 35.92 (-C=C-CH_2_-C=C-), 32.75 (-C=C-CH_2_-C=C-), 31.52 (CH_3_CH_2_-), 30.90 (-CH_2_C=O-), 29.61 (-C=C-CH_2_CH_2_-), 29.34 (-(CH_2_)_5_-, cyclohexyl), 29.26 (-(CH_2_)_5_-, cyclohexyl), 29.14 (-(CH_2_)_5_-, cyclohexyl), 27.22 (-(CH_2_)_5_-, cyclohexyl), 26.39 (-(CH_2_)_5_-, cyclohexyl), 25.62 (-(CH_2_)_5_-, cyclohexyl), 25.53 (-(CH_2_)_5_-, cyclohexyl), 25.48 (-(CH_2_)_5_-, cyclohexyl), 25.34 (-(CH_2_)_5_-, cyclohexyl), 24.73 (-(CH_2_)_5_-, cyclohexyl), 22.58 (-(CH_2_)_5_-, cyclohexyl), 20.55 (-(CH_2_)_5_-, cyclohexyl), 14.28 (-CH_3_).; HRMS (ESI+) *m*/*z* Calcd. for C_31_H_53_N_2_O_2_ + [M + H] + 485.4102 found 485.4092.

The synthesis of MSR405 is illustrated in Figure 1A. First, to solution of the α-linolenic acid (a), 45 mL Tetrahydrofuran (THF) was added with Dicyclohexylcarbodiimide (DCC) and 4-dimethylaminopyridine (DMAP). The resulting mixture was stirred at the atmosphere pressure at 45 °C for 24 h and subsequently analyzed by thin-layer chromatography (TLC) (n-heptane:ethyl acetate = 5:1) using iodine as the detection agent. Once the compound (a) was fully consumed, the solvents were concentrated under vacuum. The residues were then added to n-heptane (100 mL) to generate a white solid. The mixture was stirred for 1 h and finally filtrated. Crude products were obtained, and the column purification was performed by silica gel column chromatography using ethyl acetate/n-heptane (10:1) as eluent to yield the MSR405 (b). 

### 2.2. Animals and Grouping

Female Sprague Dawley rats (age, 5 weeks; weight, 160–190 g) were obtained from the Experimental Animal Center of Guangxi Medical University in Nanning, China. The rats were housed and fed in a standard facility with a 12 h light–dark cycle at 26 °C and a controlled humidity of 70%. Rats were fed with sufficient food and water throughout the entire research process. All experimental procedures were approved by the Animal Studies Committee of Guangxi University in Nanning, China (approval code: GXU-2024-011; approval date: 1 December 2023), and were conducted following their guidelines.

After one week of acclimatization, we divided animals into four groups randomly: Sham (*n* = 6), SCI (*n* = 6), SCI-M20 (*n* = 6), and SCI-M40 (*n* = 6). The SCI-M20 and SCI-M40 groups of rats were administered with 20 and 40 mg/kg/d of MSR405, respectively. Each group was further divided into two time-point groups: 7 and 28 days post-spinal cord injury (SCI), with 3 rats per time-point group. All animals survived the study period without experiencing any adverse effects and were included in the data analysis.

### 2.3. SCI Model Establishment and Drug Administration

Six-week-old rats, weighing 200 g, were anesthetized using 1% sodium pentobarbital (40 mg/kg) through the intraperitoneal route. Each rat was then inflicted with spinal crush injury at the T8–T9 level using a 4 cm fully straight tooth vascular clip (Dongyu, Ningbo, China) with a 0.2 mm spacer. The vertebrae laminectomies of T8–T9 vertebrae were performed using ophthalmic micro-holding needle forceps. The rat SCI model was established by applying lateral compression to the exposed spinal cord for 30 s, which was verified by induced tail spasms [20].

Animals in the sham group experienced anesthesia, skin incision, and laminectomy, except compression. Following the injury, the muscle and skin were closed, and the animal was intramuscularly administered penicillin (20,000 units). Manual bladder emptying was performed twice daily until spontaneous voiding was restored in operated rats.

MSR405 was dissolved in saline and administered intraperitoneally to the rats once daily starting from the day of surgery until the sacrifice at 20 or 40 mg/kg. In the sham and SCI groups, saline was administered to rats in place of MSR405.

### 2.4. Behavioral Tests

To evaluate the neurological function of rats, we calculated the Basso, Beattie, and Bresnahan (BBB) score [21] at 1, 3, 7, 14, 21, and 28 days following SCI. The BBB score, ranging from 0 to 21, measures hindlimb coordination and weight-bearing ability. The better motor function was shown by a higher score. Two independent blind inspectors assessed the animals for 5 min on a transparent runway and calculated the average score.

To analyze the limb weight support and limb coordination ability of 28 days after injury rats, black and red ink was applied to the forelimbs and hindlimbs, respectively, and their footprints were recorded. Subsequently, the rats were positioned on two non-overlapping white A4 papers (size: 210 mm × 596 mm) and allowed to walk on the paper-covered narrow surface. The stride characteristic, representing the vertical distance between the forelimbs and hindlimbs, was used to assess animal coordination ability. The frequency of toe dragging, which refers to the ratio between dragging and the total number of steps, was employed to evaluate the animal weight support ability.

To analyze the determine the degree of tactile sensory changes present after SCI, we used von Frey filaments (Stoelting, Wood Dale, IL, USA) with bending forces calibrated from 0.008 g to 300 g. A von Frey filament test was performed on one group of rats 7 days after the injury [22].

### 2.5. Tissue Collection and Processing

Rats were anesthetized using intraperitoneal injection of 1% sodium pentobarbital at 400 mg/kg. Subsequently, the rats were subjected to transcardial perfusion with normal saline (100 mL) followed by 4% paraformaldehyde (100 mL). The spinal cord samples were carefully extracted from 1 cm above and below the injured lesion.

For the immunohistochemical assay, the spinal cords were immersed in 4% paraformaldehyde, extracted, and then embedded in paraffin.

### 2.6. Hematoxylin-Eosin (HE) and Nissl Staining

For HE staining, the tissue paraffin sections were first dewaxed and hydrated before staining in hematoxylin for 3–5 min. The stained sections were then quickly rinsed with distilled water. The section slides were immersed in an HCl/95% alcohol (1:50) solution for differentiation for 6 s and then again rinsed with distilled water. Next, the slides were counterstained with a bluing reagent. After another wash, the slides were stained with eosin for 5 min. Tissue dehydration was carried out using a gradient of ethanol concentrations (75% ethanol, 95% ethanol, 100% ethanol), followed by clearing with xylene and sealing with a neutral mounting medium.

Nissl staining was performed on paraffin-embedded tissue sections. The first step involved dewaxing and hydration. Subsequently, the tissue sections were immersed in the staining solution for 2–5 min, and used water to wash, followed by a brief differentiation in 0.1% acetic acid solution. The differentiation reaction was terminated by rinsing in water. The extent of differentiation was monitored under a microscope. After rinsing the sections with distilled water, they were placed in an oven for drying. Lastly, the sections were treated with xylene for 10 min to attain transparency and were subsequently mounted with neutral gum.

The article quantitatively analyzed the area of tissue voids and the number of Nissl bodies. Three replicates were performed for each group.

### 2.7. Immunofluorescence Staining

After dewaxing and hydration, we applied a repair box to put the sections in containing EDTA antigen repair buffer (pH 8.0) for antigen repair. The antigen repair process was carried out in a microwave oven. Initially, the microwave was set to medium heat for 8 min until boiling, and then the sections were allowed to sit for 8 min without heat. Subsequently, the heat was switched to medium–low and maintained for 7 min. Once the sections were naturally cooled down, they were placed in PBS (phosphate-buffered saline; pH 7.4) and subjected to decolorization on a shaker for 3 cycles. Afterward, the excess PBS was removed and a BSA blocker was added with an incubation time of 30 min. Next, primary antibodies were applied to incubate the sections overnight at 4 °C. The day following, the slides used PBS to wash thrice and subsequently used corresponding secondary antibodies to incubate for 2 h at 37 °C. Finally, the slides were sealed with a self-quenching reagent. The tissue sections were stained with the following primary antibodies: CD68 (1:200, servicebio), CD86 (1:500, servicebio), CD206 (1:2000, servicebio), and IBA1 (1:200, servicebio).

### 2.8. Cell Culture and Drug Treatment

The BV2 (Procell CL-0493) cell line was generously provided by Procell Life Science & Technology Co., Ltd., Wuhan, China, and cultured in Dulbecco’s modified Eagle’s medium (DMEM) high glucose complete medium containing 10% fetal bovine serum (FBS), 1% penicillin, and 1% streptomycin. The cells were maintained at 37 °C in a 5% CO_2_ humidified air environment to maintain the resting, non-activated state of BV2 cells.

For these experiments, we divided the cells into four groups: Control, LPS, LPS-M20 (LPS + 20 μmol/L MSR405), and LPS-M40 (LPS + 40 μmol/L MSR405). MSR405 in 0.1% dimethyl sulfoxide (DMSO) was added to the cell culture medium.

### 2.9. Cell Viability Assay

The survival viability of BV-2 cells was estimated using a Cell Counting Kit-8 (CCK-8; Shanghai Saint-bio–Biotechnology Co., Ltd., Shanghai, China). Briefly, BV-2 cells in the logarithmic growth phase were seeded in a 96-well culture plate at 5 × 10^4^ cells/mL (100 μL per well). Then, the cells were treated with different concentrations of MSR405 or LPS, and the optical density (OD value) of the cell mixture was determined. Subsequently, the cells were pre-treated with different concentrations of MSR405 for 2 h, followed by stimulation with 2 μg/mL of LPS for 24 h. Afterward, 10 μL of CCK-8 solution was added to each well, and the optical density (OD value) of each well was measured at 490 nm using a microplate reader after a 3 h incubation period.

cell viability (%) = (drug groups − blank groups)/(control wells − blank wells) × 100%.

### 2.10. Nitic Oxide (NO) Assay

BV-2 microglial cells in the logarithmic growth phase were cultured in a 12-well plate. The cultured cells were divided into four groups: control, LPS, and two treatment groups with 20 and 40 μmol/mL of MSR405, respectively. Except for the control and LPS groups, BV-2 cells in the two treatment groups were pretreated with MSR405 for 2 h, followed by treatment with 2 µg/mL LPS. After 24 h of incubation, the NO content in the supernatant was measured using the NO Griess assay kit from Beyotime Biotechnology, Shanghai, China, at 540 nm using a microplate reader.

### 2.11. Western Blotting

Cellular proteins were extracted using the RIPA lysis buffer, and the protein concentrations were determined using the bicinchoninic acid (BCA) protein kit. Protein samples were resolved by sodium dodecyl sulfate-polyacrylamide gel electrophoresis (SDS-PAGE) and then transferred onto a polyvinylidene fluoride (PVDF) membrane. The membrane was incubated overnight at 4 °C with the appropriate concentration of primary antibody. The primary antibody was diluted in proportion to TLR4 (1:5000; proteintech), IKKB (1:1000; Donglin), Phospho-IκBα (1:2000; Abcam), IκB Alpha (1:2000; proteintech), NF-κB p65 (1:1000; proteintech), Phospho NF-κB p65 (1:1000 CST), TNF-α (1:1000; Abcam), IL-1β (1:1000; Abcam), IL-6 (1:1000; Abcam), and β-actin (1:1000; Beyotime). Subsequently, the PVDF membrane was washed with Tris-buffered saline with Tween 20 (TBST) and incubated with the secondary antibody at room temperature for 2 h. Finally, the bands were detected through chemiluminescence imaging utilizing a chemiluminescent imager, and protein levels were determined based on their band intensity by Image J software (FIJI).

### 2.12. Statistical Analysis

GraphPad Prism 9 was used for data analysis. All data were expressed as mean ± standard error of the mean (SEM). The significances for data were evaluated by one-way ANOVA, followed by Dunnett’s multiple comparisons test, and *p* < 0.05 was considered statistically significant.

## 3. Results

### 3.1. Structural Analysis of Compounds

(9E,12E,15E) -N-cyclohexyl-N- (cyclohexyl carbamoyl) octadeca-9,12,15-trienamide (yellow oil, yield 60%) was obtained using an enhanced drug synthesis methodology. Based on the structure and synthetic design of the reactants, the compound was identified as N,N′-dicyclohexyl-N-linolenic acid acylurea, (9E,12E,15E) -N-cyclohexyl-N- (cyclohexyl carbamoyl) octadeca-9,12,15-trienamide based on the spectral data (Figure 1).

### 3.2. MSR405 Treatment Promoted the Recovery of Motor Function in SCI Rats

**Figure 2:**
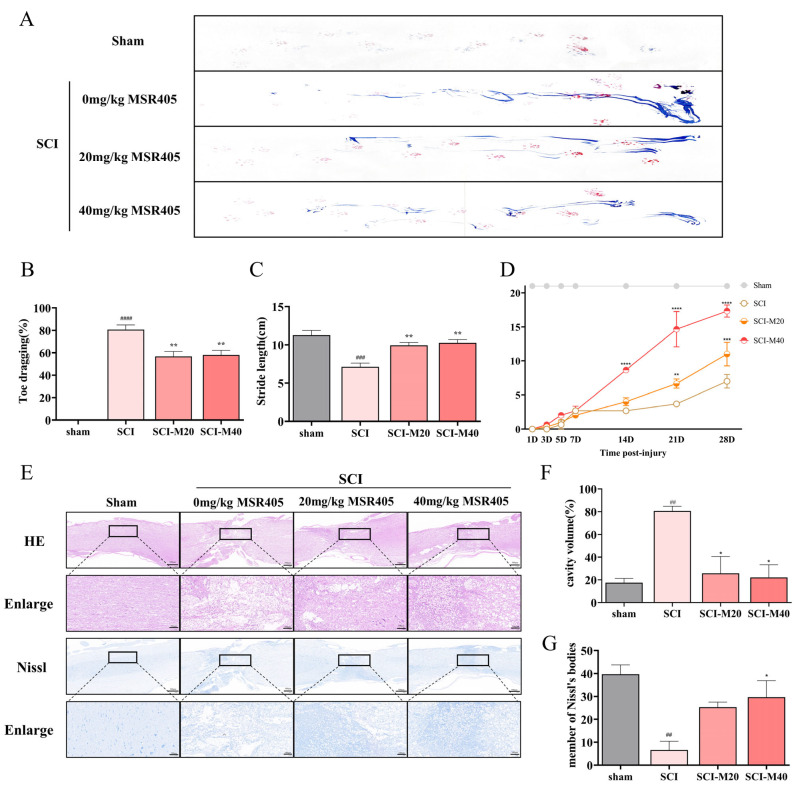
MSR405 facilitated the recovery of motor function and decreased defects of tissues. (**A**) An example picture of footprints 28 days after injury, divided into different groups. Red footprints represent forelimbs, and the blue footprints represent hindlimbs. There were no abnormal findings for the forelimb footprints, but we found that the hindlimb footprints had differences across the four groups. (**B**,**C**) Stride length, and toe dragging were used to quantify the recovery of locomotion at 4 weeks after injury (*n* = 3). (**D**) Between 0 and 28 days after injury, BBB scores were measured (*n* = 3). (**E**) We used HE staining and Nissl staining at 28 days post-operation to illustrate the histological analysis of the spinal cords of different groups. (**F**) The cavity areas were used to quantify the recovery of locomotion at 4 weeks after injury (*n* = 3). (**G**) Members of Nissl’s bodies were used to quantify the recovery of locomotion at 4 weeks after injury (*n* = 3). Data present as the mean ± SEM. ^##^ *p* < 0.01, ^###^ *p* < 0.001 vs. sham group, ^####^ *p* < 0.0001 vs. sham group, * *p* < 0.05, ** *p* < 0.01, *** *p* < 0.001 vs. SCI group, **** *p* < 0.0001 vs. SCI group.

Behavioral experiments were performed to assess the functional recovery of SCI post-MSR405 treatment. All rats underwent footprints and Basso, Beattie, and Bresnahan locomotor rating scale (BBB scale) analyses until sacrificed for further tests. In the footprint analysis, the rats in the treatment group exhibited fairly consistent posterior limb coordination. In contrast, SCI group animals showed extensive foot-dragging (Figure 2A). Furthermore, we recorded the stride length, too. On the one hand, rats in the high/low-dose treatment group showed higher stride values. On the other hand, rats in the SCI group exhibited a significant difference with high/low-dose treatment group. (Figure 2B). There similar results were obtained in the toe-dragging analysis (Figure 2C). In the first week following SCI, no noticeable differences were observed among the groups. However, over the subsequent two weeks, rats in the high-dose group displayed the most rapid recovery, which gradually slowed down thereafter. By the fourth week, a significant difference was shown in the low-dose group compared with the SCI group (saline-treated rats). The BBB scores, 4 weeks after SCI, were 17.33, 11.00, and 7.00 in the SCI-M40, SCI-M20, and SCI groups, respectively (Figure 2D). These findings indicated that rats in the high/low-dose group experienced a substantial improvement in post-SCI motor dysfunction compared to the SCI group.

### 3.3. MSR405 Attenuated Neurological Damage

To illustrate the overall morphology of the tissue, the HE staining of spinal pathological slices was performed 4 weeks after surgery. Slices in the SCI-M20 and SC-M40 groups showed less degeneration and a more organized tissue arrangement (Figure 2E), cavity area was reduced (Figure 2F). Regarding Nissl staining, we observed that both the MSR405 treatment groups exhibited a greater number of Nissl bodies compared to the SCI group (Figure 2E,G). The studies which we mentioned recommended that MSR405 substantially reduced tissue inflammation and alleviated SCI in rats.

### 3.4. MSR405 Inhibited M1 Microglia Polarization in SCI Rats

**Figure 3:**
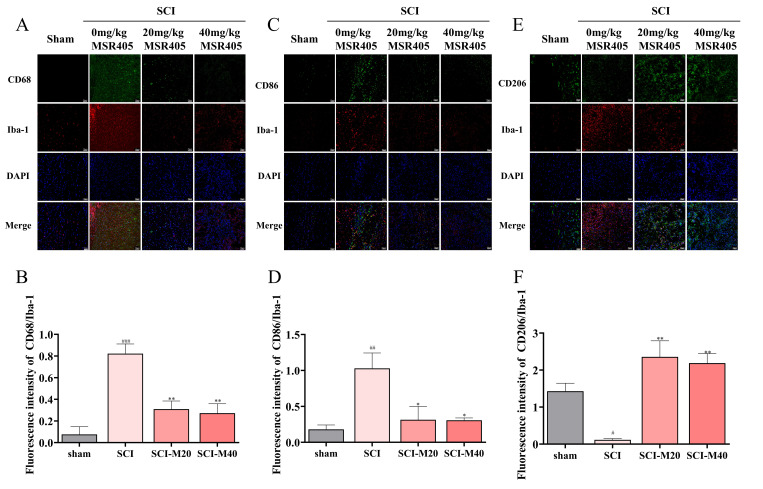
MSR405 attenuates microglial inflammation after SCI. (**A**,**B**) Double-fluorescence staining and statistical analysis for Iba-1 (red)/CD68 (green)/DAPI (blue) in spinal cord tissue sections at 7 days after SCI. (**C**,**D**) Double-fluorescence staining and statistical analysis for Iba-1 (red)/CD86 (green)/DAPI (blue) in spinal cord tissue sections at 7 days after SCI. (**E**,**F**) Double-fluorescence staining and statistical analysis for Iba-1 (red)/CD206 (green)/DAPI (blue) in spinal cord tissue sections at 7 days after SCI; *n* = 3 per group. The data are presented as the mean ± SEM. ^#^ *p* < 0.05, ^##^ *p* < 0.01, ^###^ *p* < 0.001, vs. sham group; * *p* < 0.05, ** *p* < 0.01, vs. SCI group.

Treatment with MSR405 reduced spinal cord inflammation, modulated microglia polarization, and promoted recovery from SCI in rats. Microglial activation plays a pivotal role in neuroinflammation following SCI. CD68 and CD86 were identified as markers of the M1 phenotype, and CD206 was identified as a marker of the M2 phenotype. We used CD68/Iba-1 double-staining to investigate the potential role of MSR405 in microglial activation. We observed a decrease in CD68-positive antibodies 7 days post-SCI in MSR405-treated rats compared to SCI rats. (Figure 3A,B). Along these lines, we also noticed a decrease in CD86-positive antibodies (Figure 3C,D) and increased CD206-positive antibodies 7 days after SCI in MSR405-treated rats compared to saline-treated rats (Figure 3E,F).

### 3.5. Effects of MSR405 on BV-2 Cell Viability

**Figure 4:**
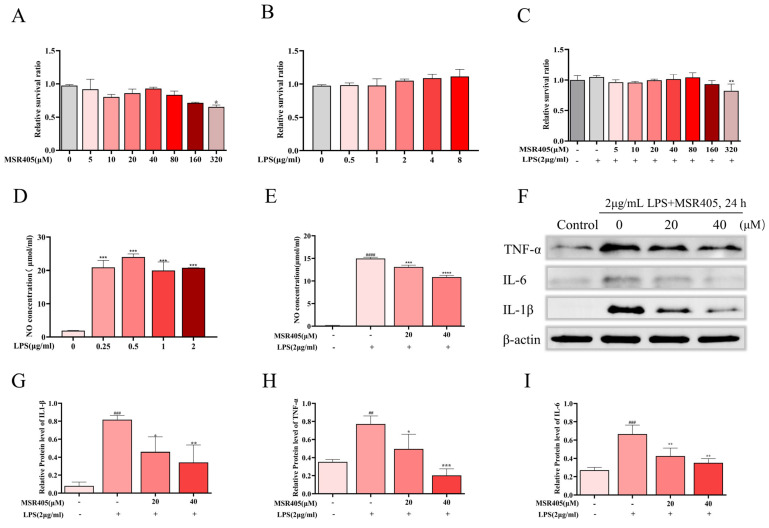
Effects of MSR405/LPS on the survival viability of BV-2 cells and MSR405 inhibited NO expression in BV-2 cells. (**A**) Influence of MSR405 on cell survival rates. (**B**) Influence of LPS on cell survival rates. (**C**) Influence of MSR405 pre-cultured on the survival rate of cells stimulated by LPS. (**D**,**E**) Influence of MSR405 on the NO concentration in the supernatant stimulated by LPS. (**F**–**I**) Influence of MSR405 on the levels of TNF-α, IL-6, and IL-1β by LPS. Different concentrations of MSR405 were used to culture BV-2 cells for 2 h and then utilized the LPS (2 µg/mL) for 24 h to incubate the BV-2 cells; *n* = 3 per group. The data are presented as the mean ± SEM. ^##^ *p* < 0.01, ^###^ *p* < 0.001, ^####^ *p* < 0.0001 vs. control group; * *p* < 0.05, ** *p* < 0.01, *** *p* < 0.001, **** *p* < 0.0001 vs. LPS group.

To assess the cytotoxicity of MSR405 on BV-2 cells, the CCK-8 reagent was used to describe their survival viability after treatment with varying concentrations of MSR405 (5, 10, 20, 40, 80, 160, and 320 μmol/mL) (Figure 4A) Additionally, we found that LPS (2 µg/mL) did not affect cell viability (Figure 4B). Furthermore, we used varying concentrations of MSR405 to culture BV-2 cells for 2 h, and then utilized the LPS (2 µg/mL) for 24 h to incubate the BV-2 cells. We noticed that the stimulation of LPS had no impact on the BV-2 cells at 5, 10, 20, 40, 80, 160, and 320 μmol/mL MSR405, which were pre-treated (Figure 4C).

### 3.6. MSR405 Suppressed LPS-Induced NO Production and Inflammatory Response in BV2 Cells

BV-2 cells were incubated with varying concentrations of LPS for 24 h. Initially, the effects of LPS-induced inflammatory mediators were evaluated by assessing NO production. We observed a significant increase in NO release from BV-2 cells after LPS stimulation; 2 µg/mL LPS produced the most consistent results (Figure 4D). Subsequently, we used varying concentrations of MSR405 to culture BV-2 cells for 2 h and then utilized the LPS (2 µg/mL) for 24 h to incubate the BV-2 cells. In the MSR405 group, the release of NO showed a significant increase after LPS stimulation of BV-2 cells, while the MSR405-treatment groups displayed a notable reduction (Figure 4E). The analysis of Western blot showed that MSR405 had regulatory effects on inflammatory cytokines. The results revealed that LPS stimulation substantially elevated the protein levels of TNF-α, IL-1β, and IL-6, whereas MSR405 pretreatment significantly alleviated them (Figure 4F–I). These results indicated that MSR405 is a significant inhibitor of LPS-induced inflammation in BV-2 cells.

### 3.7. Effects of MSR405 on LPS-Induced Polarization of BV-2 Cells

**Figure 5:**
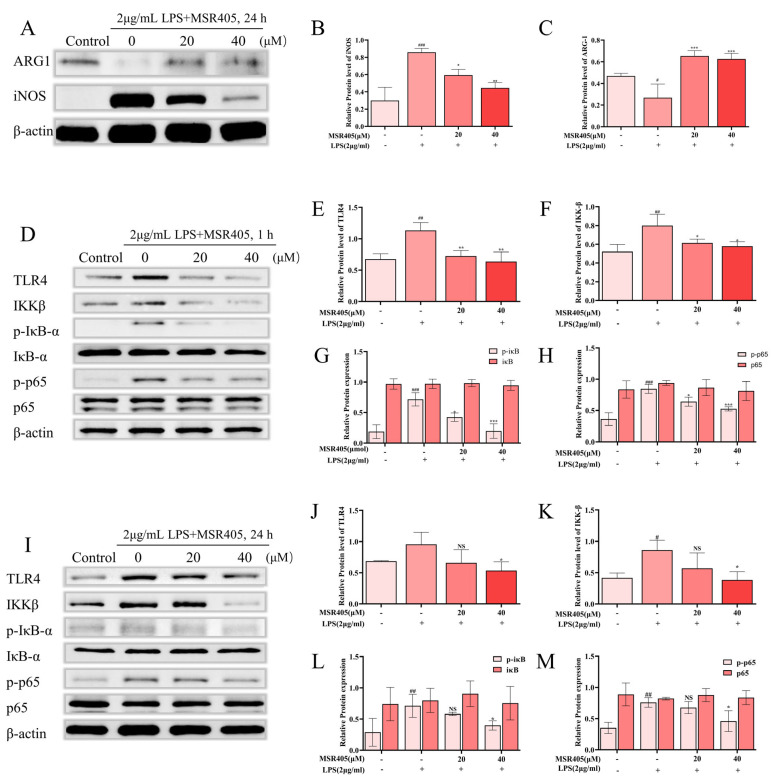
Expression of iNOS, Arg-1, and TLR4/NF-κB signaling in BV-2 cells. (**A**–**C**) Varying concentrations of MSR405 were used to culture BV-2 cells for 2 h and then utilized the LPS (2 µg/mL) for 24 h to incubate the BV-2 cells; the expression of Western blotting. (**D**–**H**) Varying concentrations of MSR405 were used to culture BV-2 cells for 2 h and then the LPS (2 µg/mL) was utilized for 1 h to incubate the BV-2 cells; the expression of Western blotting. (**I**–**M**) Varying concentrations of MSR405 were used to culture BV-2 cells for 2 h and then the LPS (2 µg/mL) was utilized for 24 h to incubate the BV-2 cells; the expression of Western blotting. *n* = 3 per group. The data are presented as the mean ± SEM. ^#^ *p* < 0.05, ^##^ *p* < 0.01, ^###^ *p* < 0.001, vs. control group; * *p* < 0.05, ** *p* < 0.01, *** *p* < 0.001 vs. LPS group. NS, no significance.

To investigate whether the protective effects of MSR405 on LPS-stimulated BV-2 cells were associated with polarization, we used Western blotting to estimate the change in the levels of M1/M2 polarization markers, namely iNOS and Arg-1. Following a 24 h pre-treatment with MSR405, iNOS expression was significantly suppressed, while that of Arg-1 was enhanced in LPS-stimulated BV-2 cells (Figure 5A–C). These results strongly indicated that MSR405 inhibited the LPS-induced M1 polarization in BV-2 cells, shifting it towards the M2 phenotype.

### 3.8. MSR405 Reduces Microglia Activation via the TLR4/NF-κB Signaling Pathway

LPS recognition functions through the TLR4/NF-κB signaling pathway [23]. By using Western blotting, we estimated the MSR405 impact of TLR4/NF-κB signaling pathway. As shown in Figure 5D–H, after 1 h (short-term) of LPS stimulation, the protein levels of TLR4, Phospho-IκB-α, IKK-β, and Phospho-p65 in the MSR405 treatment groups were significantly downregulated compared to the control group. This result indicated that in short-term LPS induction, pre-culturing with MSR405 effectively lowered the levels of TLR4, Phospho-IκB-α, IKK-β, and Phospho-p65. However, following 24 h of LPS induction, we found no significant differences in levels of the above-mentioned proteins between the low-dose MSR405 group and the control group (Figure 5I–M).

## 4. Discussion

SCI, a severe traumatic event, profoundly affects the central nervous system (CNS) [24]. The pathophysiology of SCI is intricately linked to the inflammatory response, which directly influences a patient’s recovery and functional outcomes [25]. The long-term chronic inflammation of SCI stimulates the excessive activation of motor neurons and glial cells, leading to central nervous system (CNS) dysfunction and the formation of scar tissue, which hinders SCI repair [26]. The central therapeutic strategy for SCI involves curtailing the excessive expression of pro-inflammatory cytokines in the CNS [27].

The commonly used animal models for spinal cord injury include rats, mice, pigs, dogs, cats, primates, etc. The most frequently used animal model is the rat. Common experimental methods include spinal cord contusion, while other less clinically relevant models include transection or hemitransection. In this study, we used a simple and inexpensive method of producing an experimental compressive model of spinal cord injury in rats by means of a vascular clip. This model of spinal cord compression injury causes a standardized injury, as well as efficient and reproducible functional and morphological outcome measurements [28].

Microglia, the native immune cells of the central nervous system, play an important role in the neuroinflammatory response. The overactivation of microglia is recognized as one of the key factors in neuroinflammation and reactive gliosis. Microglia can be divided into two major subsets: proinflammatory (M1) (classically activated) microglia and anti-inflammatory (M2) (alternatively activated) microglia. Decreasing M1 microglial polarization can reduce neuroinflammation and ameliorate neuromotor dysfunction after SCI [29]. It is well established that SCI prompts the activation of microglia towards the M1 phenotype [30]. This heightened activation accelerates the release of inflammatory cytokines, such as IL-1β, IL-6, and TNF-α [31], triggering a post-spinal cord injury inflammatory cytokine storm and accelerating neuronal cell apoptosis [32,33]. Therefore, it is crucial to suppress this overexpression of inflammatory cytokines to manage secondary injury after SCI [34].

Research has demonstrated the significant activation of the TLR4/NF-κB signaling pathway and a substantial increase in the inflammatory response following SCI [35]. Nuclear factor-κB (NF-κB) represents TLR4’s downstream molecule, which can be activated by TLR4. The TLR4/NF-κB pathway blockage is able to enhance SCI mouse functional recovery and reduce SCI-induced secondary injury (such as apoptosis and inflammatory response) [36]. Inflammatory factors and infections stimulate the NF-κB signaling pathway, activating microglia [37]. The activation of NF-κB promotes the expression of downstream target genes that regulate microglial localization and activation in the afflicted region [38]. Furthermore, it also regulates the expression of inflammatory mediators, influences macrophage differentiation, and determines cell fate [39]. Growing evidence indicates that NF-κB activation following SCI stimulated microglia to polarize towards M1 [40].

*Radix Isatidis*, a member of the Cruciferae family [41], has anti-inflammatory properties [42] and is recognized for its low toxicity and minimal side effects [17]. Hence, we selected *Radix Isatidis* as a foundational compound to optimize its structure and develop a range of acyl urea compounds. In this study, we improved the synthesis process of MSR405 to increase its yield. Additionally, mass spectrometry analysis confirmed the substantial improvement in sample purity. Our experiments demonstrated that MSR405 effectively enhanced motor function in SCI rats, accelerated post-injury recovery, and facilitated nerve repair Seven days after injury, and apart from some recovery regarding pain reflex, rats in the SCI group exhibited no significant differences to the high/low-dose treatment groups. Perhaps it is because within seven days of spinal cord injury, the rats were still in the acute and subacute phase, and the lower limb sensations of the rats had not yet recovered. In future research, it will be important to explore the sensory recovery of spinal cord injury in rats treated with MSR405 over extended time periods.Additionally, we uncovered the inhibitory effect of MSR405 on inflammatory factors in the spinal cord. And MSR405 has low cytotoxicity and excellent stability.

Through in vitro experiments, we further investigated the anti-inflammatory properties of MSR405. We found that MSR405 treatment significantly reduced the expression of pro-inflammatory factors, such as TNF-α, IL-1β, and IL-6, in BV-2 cells that were exposed to LPS for 24 h. Additionally, MSR405 treatment significantly decreased the expression of iNOS, a marker protein for M1 polarization, while upregulating ARG-1, a marker protein for M2 polarization. These results suggested that MSR405 facilitates M1 to M2 transition in BV-2 cells, which may contribute to its anti-inflammatory effects.

NF-κB, a transcription factor, binds to the enhancer of the immunoglobulin κB light chain gene and exerts crucial regulatory roles across diverse cells through multiple signaling pathways [43]. Particularly within the context of inflammatory reactions, NF-κB performs a vital role [44] in regulating the transcription, phosphorylation, and subsequently nuclear translocation of inflammatory factors [45]. In our in vitro experiments, we found that within a span of 1 h, MSR405 effectively suppressed the levels of proteins consisted with the NF-κB signaling pathway. However, after 24 h, apparent differences in protein expression were observed only in the high-dose group, indicating the concentration-dependent effect of MSR405 treatment.

**Figure 6:**
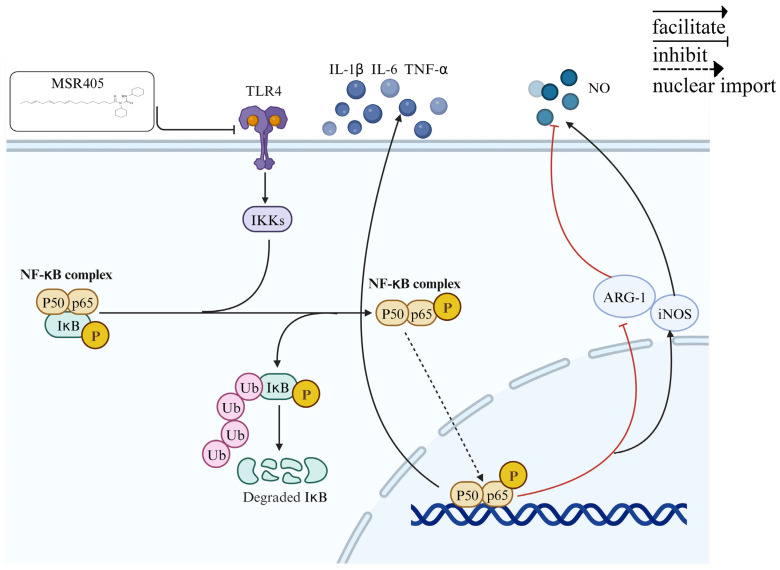
Schematic representation of molecular mechanisms underlying the inhibitory effect of MSR405 on Neuroinflammation.

Overall, our data suggested that MSR405 can inhibit neuroinflammation after SCI in rats by modulating the TLR4/NFKB signaling cascade to alter the activation state of microglia. Although MSR405 inhibits the M1 polarization of microglia, the effects of MSR405 on other CNS members have not been clarified, which is necessary for future studies. Our results, to some extent, uncover the underlying molecular mechanism of MSR405 in neuroprotection against SCI (Figure 6) and provide theoretical groundwork for potential clinical applications.

MSR405, as a newly developed drug, on one hand, compared to the previously published herbal monomers, MSR405, as a newly synthesized derivative of *Radix Isatidis* unsaturated fatty acids, differs in its chemical structure and therapeutic mechanism, providing novel approaches and methods for SCI treatment. Our research has demonstrated that MSR405 can inhibit proinflammatory cytokines, support anti-inflammatory responses, and block the TLR4/NF-κB signaling pathway. This well-defined mechanism may render MSR405 more targeted and effective in treating SCI. On the other hand, although the efficacy of MSR405 was initially validated in rat models of SCI, the current synthesis and production of MSR405 may involve higher costs, limiting its widespread application, and we will continue to explore its mechanisms in subsequent experiments.

Similar homologous compounds have been administered via gavage for other diseases and have shown significant effects (such as *N*,*N*′-dicyclohexyl-*N*-arachidonic acylurea, α-Linolenic acid) [46,47]. In our study, we used intraperitoneal injections, which showed no first pass eliminations and higher fractions of bioavailability compared to gavage. Therefore, we speculate that MSR405 has value for further research. At present, our experimental results have preliminarily verified the inhibitory effect of MSR405 on neuroinflammation. We will further explore the pharmacokinetics of MSR405 in future research.

As a newly developed drug, the potential impact of MSR405 has not been fully validated. This study only indicated the good anti-inflammatory effect of MSR405 in in vitro experiments, lacking its impact on in vivo cytokines. In future research, we will use MRI to better detect the clinical response of MSR405 to SCI treatment, as well as the sampling of cells and chemotactic genes. We will track the recovery of sensory recovery over a longer period of time to further refine the argument in this article.

## Data Availability

The raw data supporting the conclusions of this article will be made available by the authors on request.
